# Comparison of prognostic outcomes in vitrectomy for myopic foveoschisis with foveal detachment: air tamponade versus no-air tamponade

**DOI:** 10.1186/s40942-026-00884-5

**Published:** 2026-06-27

**Authors:** Juan Zhang, Hao Chang, Shixue Liu, Xin Huang, Qing Chang

**Affiliations:** 1 Department of Ophthalmology, Eye and ENT Hospital of Fudan University, Shanghai, 200031 China; 2 Shanghai Key Laboratory of Visual Impairment and Restoration, Shanghai, China; 3 NHC Key Laboratory of Myopia and Related Eye Diseases, Chinese Academy of Medical Sciences, Shanghai, China; 4 Eye Institute and Department of Ophthalmology, Eye and ENT Hospital, Fudan University, No. 83, Fenyang Road, Xuhui District, Shanghai, 200030 China

**Keywords:** Myopic foveoschisis, Foveal detachment, Pars plana vitrectomy, Air tamponade, Anatomical outcomes, Visual outcome

## Abstract

**Purpose:**

To compare the efficacy of pars plana vitrectomy with or without air tamponade for patients with myopic foveoschisis (MFS) and foveal detachment (FD).

**Methods:**

Forty-seven patients (47 eyes) with MFS and FD who underwent PPV with fovea-sparing internal limiting membrane peeling were included in this retrospective analysis. Patients were divided into air-tamponade (*n* = 21) and no-air-tamponade (*n* = 26) groups. Central foveal thickness (CFT), external limiting membrane (ELM) / ellipsoid zone (EZ) integrity, best-corrected visual acuity (BCVA), and incidence of complications were compared.

**Results:**

In longitudinal analysis, the air-tamponade group had significantly faster CFT reduction at 1 week and 1 month (both *P* < 0.05). By 3 and 12 months, the CFT values were comparable between groups. Time to foveal reattachment was shorter with air tamponade (*P* < 0.001). The ELM and EZ integrity were compared at 12 months (both *P* > 0.05). Postoperative BCVA showed significant improvement from baseline at 3 and 12 months in both groups (*P* < 0.05). The time-by- tamponade type interaction at 3 and 12 months was not significantly different on BCVA in multivariable model (*P* > 0.05). Baseline diameter of FD was identified as an independent risk factor of 12-month BCVA by multivariable analysis. Complication rates, including postoperative macular hole, macular hole with retinal detachment, and transient aggravation, did not significantly differ between groups.

**Conclusion:**

Air tamponade accelerated early anatomical recovery in MFS with FD, but both tamponade types achieved comparable long-term anatomical and visual outcomes. Baseline FD diameter was the independent predictor of visual outcome.

**Clinical trial number:**

Not applicable.

**Supplementary Information:**

The online version contains supplementary material available at https://doi.org/10.1186/s40942-026-00884-5.

## Key messages

**What is known:**

• The surgical treatment for myopic foveoschisis (MFS) with foveal detachment (FD) has been challenging, and the effects of intraoperative air tamponade remain uncertain.

**What is new:**

• The use of air tamponade significantly accelerated anatomical recovery in MFS with FD compared to no-air tamponade, but both groups presented comparable visual outcomes and post-operative macular hole rates.

• Preoperative diameter of FD was independently associated with visual outcomes.

**Supplementary Information:**

The online version contains supplementary material available at https://doi.org/10.1186/s40942-026-00884-5.

## Introduction

Pathologic myopia is a major public health concern and one of the leading causes of irreversible vision loss globally [1–3]. It is defined by excessive axial elongation and formation of posterior staphyloma, which induces a series of progressive and vision-threatening structural changes in the posterior segment of the eye [4, 5]. Among these changes, myopic traction maculopathy (MTM) comprises a spectrum of complications, including retinoschisis, macular hole (MH), and foveal detachment (FD), which represents a significant cause of visual deterioration in highly myopic eyes [6, 7].

Myopic macular retinoschisis constitutes a primary component of MTM, of which myopic foveoschisis (MFS) may lead to tractional FD and has been recognized as a primary contributor to visual loss in patients with pathologic myopia [8, 9]. Pars plana vitrectomy (PPV) remains the standard surgical method for MFS and FD, aiming to release vitreoretinal traction, restore retinal architecture, and prevent additional deterioration [10–12]. After performing PPV and internal limiting membrane (ILM) peeling, surgeons may elect to employ a gas tamponade within the vitreous cavity to promote the reattachment of the detached foveal retina [13]. However, because of the thin anatomic structure in MFS and FD, postoperative MH occurs in 5%27.3% of eyes receiving PPV with complete ILM peeling [14–17]. Recently, PPV with fovea-sparing ILM peeling (FSIP) has been developed, effectively decreasing the incidence of post-operation MH [17–19].

However, the use of gas tamponade as a surgical option remains to be a subject of debate. Several studies have suggested that intraocular gas tamponade can accelerate the resolution of MFS and potentially improve functional outcomes [20, 21]. Conversely, other reports have demonstrated successful anatomical recovery and even favorable visual outcomes without the use of gas tamponade, thereby avoiding gas-related complications and discomfort [22, 23]. A recent meta-analysis indicated that intraocular long-acting gas tamponade with sulfur hexafluoride (SF_6_) or perfluoropropane (C_3_F_8_) may lead to increased rates of gas-related complications and postoperative MH compared with air tamponade [13], which may be attributed to the comparatively weaker mechanical stress of air on the thin fovea. However, the surgical efficacy of intraocular air tamponade versus fluid-only tamponade in vitrectomy for MFS and FD has not been clarified.

Therefore, we conducted a retrospective comparative study to assess the anatomical and visual outcomes following vitrectomy with versus without intraocular air tamponade in eyes with MFS associated with FD.

## Methods

### Patient enrollment

**Fig. 1:**
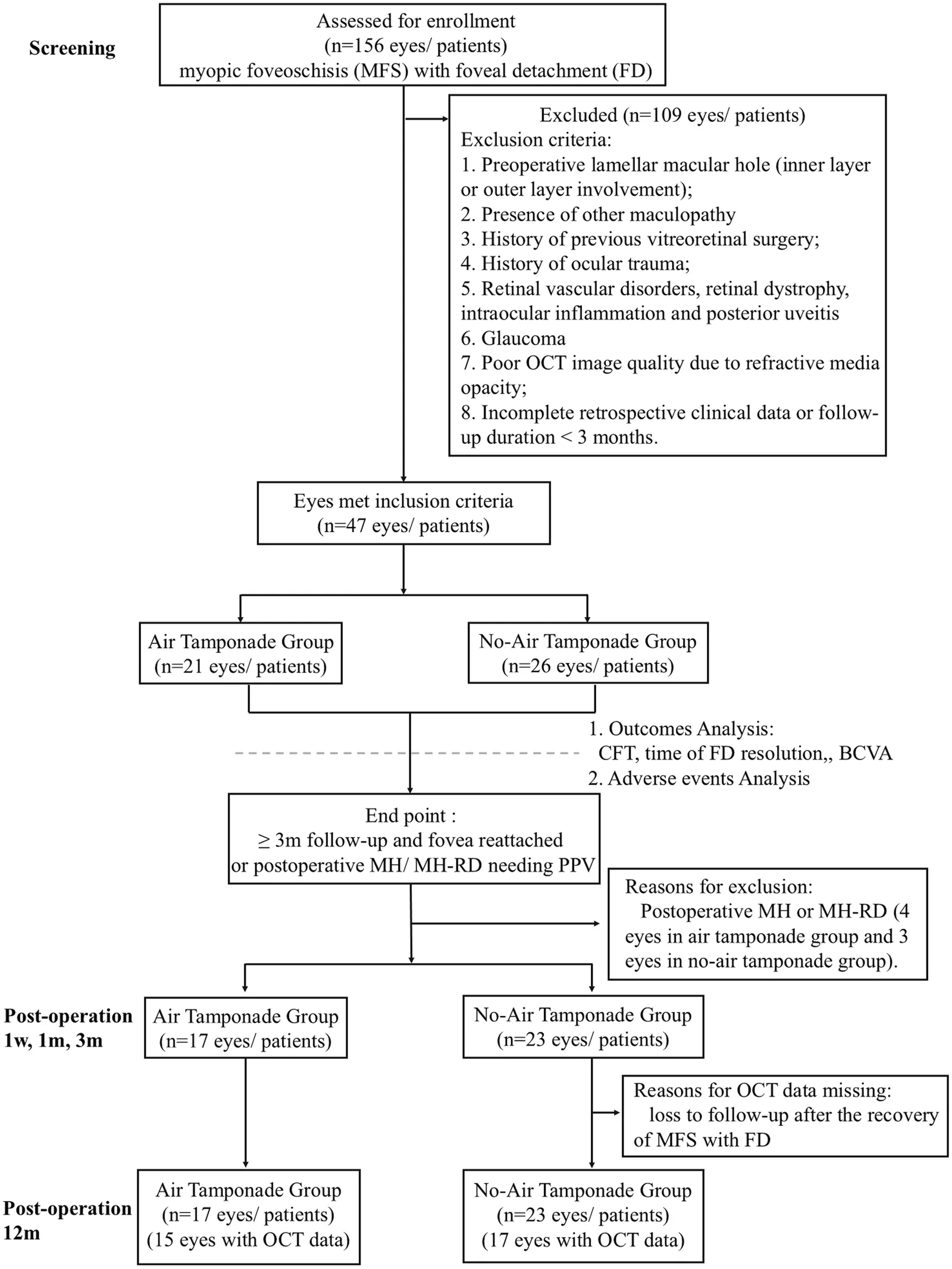
The flow diagram of this retrospective study. The detailed information of exclusion criteria, outcomes and data missing

This retrospective study was conducted in accordance with the Declaration of Helsinki and received approval from the Institutional Review Board of the Eye and ENT Hospital at Fudan University. A total of 156 eyes from patients with MFS who underwent PPV at our institution and were aged over 18 years between 2017 and 2022 were screened. Among these, 47 eyes were eligible and enrolled in the analysis. The following inclusion criteria were established: (1) high myopia with an axial length > 26.5 mm, (2) MFS accompanied by FD, (3) receiving PPV with FSIP, and (4) a postoperative follow-up period of no less than 3 months. The exclusion criteria included (1) preoperative lamellar MH only (inner layer or outer layer involvement) and full-thickness MH; (2) the existence of other maculopathy (e.g., fibrotic scar, choroidal neovascularization or age-related macular degeneration); (3) History of previous vitreoretinal surgery; (4) History of ocular trauma; (5) Retinal vascular disorders (e.g., diabetic retinopathy or retinal vascular occlusion), retinal dystrophy, intraocular inflammation, posterior uveitis; (6) Glaucoma; (7) Patients with incomplete retrospective clinical data or follow-up duration < 3 months; (8) Poor OCT image quality due to refractive media opacity. Patients were followed up monthly until either their fovea reattached or postoperative complications needing further surgery, including MH and retinal detachment (RD), occurred. The flow diagram was shown in Fig. 1. All enrolled participants signed informed consent for use of their data in this manuscript.

### Data collection and treatment

Baseline demographic and clinical information was documented, including age, sex, axial length (IOLMaster, Carl Zeiss Meditec, Jena, Germany), and lens status. The best-corrected visual acuity (BCVA) was assessed using Snellen charts and subsequently converted into logarithms of the minimum angle of resolution (logMAR). Visual change exceeding 0.2 logMAR between postoperative and preoperative BCVA served as the threshold for defining visual improvement or deterioration. We also recorded the preoperative intraocular pressure (IOP) and findings of optical coherence tomography (OCT, Heidelberg Engineering, Heidelberg, Germany), including diameter of FD, presence of inner or outer foveoschisis, central foveal thickness (CFT), and neurosensory CFT (NCFT). CFT was measured as the vertical distance fromthe ILM to the retinal pigment epithelium in the center of fovea. NCFT was defined as the thickness of the neurosensory retina, measured from the ILM to the outer surface of the photoreceptor layer at the foveal center. The diameter of FD was quantified as the mean value of the horizontal and vertical diameters measured on enface OCT images. The integrity of the outer retinal layers, including external limiting membrane (ELM) and the ellipsoid zone (EZ), was assessed within a 1-mm-diameter area centered on the fovea. Based on the grading system validated by dell’Omo et al. [24], the layer integrity was classified using a 4-point grading scale: Grade 1, line not visible; Grade 2, line disruption > 200 μm; Grade 3, line disruption < 200 μm; and Grade 4, continuous line (Supplemental Fig. 1). These OCT parameters were independently measured by two experienced ophthalmologists (JZ and HC) masked to clinical data and group allocation, utilizing the built-in caliper tool of the OCT equipment. The mean value was used for analysis. Any discrepancy exceeding a predefined threshold (10%) was resolved by a third senior grader (QC).

All patients received PPV with a conventional 23- or 25-gauge system. The surgical procedure comprised a complete vitrectomy and FSIP. Triamcinolone acetonide and indocyanine green were utilized for ILM peeling. Following vitrectomy, the vitreous cavity was filled with either air (*n* = 21) via fluidair exchange or balanced salt solution (*n* = 26). At the same time, all phakic patients received combined phacoemulsification along with intraocular lens implantation. The decision to use air tamponade versus no-air tamponade was based on the time period: air tamponade was performed from 2017 to 2019, and no-air tamponade was used thereafter.

### Outcomes

The primary outcome was anatomical recovery, including anatomical improvement of FD (as indicated by postoperative CFT reduction), rate of FD resolution, posterior ELM / EZ integrity. Other outcomes included postoperative BCVA, IOP change, and incidence of postoperative complications (e.g., MH formation, MH-related RD, transient aggravation, and choroidal leakage). Eyes that developed combined MH or RD requiring reoperation were excluded from quantitative analyses of CFT and BCVA to eliminate bias due to complications. Transient aggravation was defined as a temporary worsening of macular morphology, which could be evaluated by temporary increase in postoperative CFT values compared to preoperative levels. Postoperative temporary choroidal leakage manifested as wavy changes of the choroid, retinal pigment epithelium (RPE) and neuroretina on postoperative OCT images, accompanied by unclear refractive media due to the leakage (Supplemental Fig. 2). Postoperative OCT data were collected at 1 week, 1 month, and 3 months. CFT and BCVA at 12 months were also analyzed in patients with long follow-ups.

### Statistics

Descriptive analysis of the study cohort’s characteristics and evaluation of baseline balance were conducted prior to the study. Continuous variables were summarized as the mean ± standard deviation (SD) or median with interquartile range (IQR) for non-normally distributed continuous variables, and categorical variables were presented as numbers and percentages. The distribution normality of continuous variables was assessed via ShapiroWilk test. Between-group comparisons of continuous variables were assessed using the independent-samples t-test or Wilcoxon rank-sum test, depending on the normality and homogeneity of variance assessed via Levene’s test.

This study compared the measurements of MFS with FD under different treatments (Air tamponade vs. No-air tamponade) at each follow-up time point during the study period using the t-test or the Wilcoxon test. The efficacy of two treatments was assessed in longitudinal analyses using mixed-effect models that allowed for missing data for continuous variables. This study initiated a longitudinal analysis of the efficacy of treatments, examining the effects of time, treatment, and their interaction in the crude model. Covariates including age, gender, axial length, diopter sphere, lens status and foveal morphology were then included in multivariable mixed-effect models, followed by a stepwise regression calculating the estimates with 95% confidence interval (CI) to identify the estimated efficacy of sound treatment as well as the effect of influential risk predictors. The longitudinal analyses of categorical variables were performed using generalized estimated equation (GEE), and the odds ratios (OR) with 95% CI and corresponding *P*-values were calculated. The goodness of model fitness was assessed with the Akaike Information Criterion (AIC). The random effect of the models was presented by the Intraclass Correlation Coefficient (ICC), which presented the proportion of total variance attributable to between-subject variability.

The rates of FD resolution between the groups were compared using survival analysis (Cox proportional hazards model), hazard ratio (HR) and 95% CI were also calculated. Considering the risk of increasing type I error while performing multiple comparisons, analyses on secondary outcomes were reported with effect size. The variation in treatment efficacy was estimated using different models, and models with different covariates were compared in sensitivity analysis. All analyses were performed using the statistical software RStudio (Version 2023.06.0-421; Posit Software, PBC, Boston, MA). The main longitudinal CFT/BCVA analyses were performed in the per-protocol (PP) population after excluding eyes that developed postoperative MH or MH-RD. *P*-values < 0.05 were considered significant.

## Results

### Baseline characteristics

A total of 47 eyes from 47 patients with MFS and FD were enrolled in the final analysis. Of these, 21 eyes (21 patients) underwent PPV with air tamponade, whereas 26 eyes (26 patients) underwent PPV without tamponade. Baseline characteristics between the two groups were comparable (Table 1). There were no significant differences observed in demographic parameters, including age, sex, axial length, diopter sphere, and lens status.

Preoperative BCVA (1.00 (0.70, 1.30) vs. 0.96 (0.70, 2.00), logMAR) was also similar between the two groups, ensuring comparable visual function at baseline (*Effect size* = 0.087; Small). Furthermore, the anatomical variables of the two cohorts were largely homogeneous. All eyes exhibited both external retinoschisis and FD on preoperative OCT, and the presences of inner and outer retinoschisis were consistent. Quantitative OCT parameters, including preoperative CFT, NCFT, the FD diameter, the distributions of ELM integrity and EZ integrity also showed no significant differences between the two groups (Table 1).

**Table 1:** The baseline characteristics between eyes of MFS with foveal detachment underwent vitrectomy with or without air tamponade

Characteristic	Air Filling Status				
	Air Tamponade *N* = 21	No-Air Tamponade *N* = 26	Overall *N* = 47	Effect size	
				*Effect size* ^#^	Magnitude
**Age (Mean ± SD)**	58.38 ± 10.15	57.35 ± 12.12	57.81 ± 11.18	0.093	Negligible
**Gender**				0.019	
Female	15 (71%)	17 (69%)	32 (70%)		
Male	6 (29%)	9 (31%)	15 (30%)		
**Axial Length (mm)**	29.99 ± 1.39	29.72 ± 1.62	29.84 ± 1.51	0.309	Small
**Diopter Sphere (D)**	-14.8 ± 2.4	-13.5 ± 4.4	-14.1 ± 3.7	-0.412	Small
**Lens status**				0.176	
Phakic	17 (81%)	25 (96%)	42 (89%)		
Pseudophakic	4 (19%)	1 (3.8%)	5 (11%)		
**Anatomical status**					
Inner retinoschisis	12 (57%)	19 (73%)	31 (66%)	0.122	
Outer retinoschisis	21 (100%)	26 (100%)	47 (100%)	/	
Foveal detachment	21 (100%)	26 (100%)	47 (100%)	/	
**BCVA (logMAR)** ^**2**^	1.00 (0.70, 1.30)	0.96 (0.70, 2.00)	1.00 (0.70, 1.70)	0.087	Small
**Diameter of foveal detachment (µm)** ^**2**^	902.27 (665.00, 1347.99)	929.70 (580.41, 1520.39)	919.20 (603.03, 1381.36)	0.062	Small
**CFT (µm)** ^**2**^	521.00 (424.00, 595.00)	609.09 (517.44, 685.00)	557.82 (461.04, 645.16)	0.265	Small
**NCFT (µm)** ^**2**^	95.30 (75.71, 151.56)	79.28 (49.07, 118.84)	86.21 (54.40, 131.00)	0.215	Small
**ELM** (Grade 1 / 2 /3 / 4)	16 / 5 / 0 / 0	14 / 9 / 1 / 2	30 / 14 / 1 / 2	0.253	Small
**EZ** (Grade 1 / 2 /3 / 4)	17 / 4 / 0 / 0	16 / 8 / 2 / 0	33 / 12 / 2 / 0	0.223	Small

Abbreviation: BCVA= Best Corrected Visual Acuity; CFT = central foveal thickness; NCFT = neurosensory central foveal thickness; ELM = external limiting membrane; EZ = ellipsoid zone

Statistics:

^1^Normally distributed continuous variables are presented as mean ± standard deviation (SD), ^2^Non-normally distributed continuous variables are presented as median with interquartile (IQR)

#effect size: ^1^using t-test for normally distributed continuous variables (or ^2^Wilcoxon rank-sum tests for non-normally distributed continuous variables) and Wilcoxon rank-sum tests for ordinal categorical variables (ELM grades and EZ grades) for descriptive comparison, and the difference were presented with Cohen’s d as the effect size for t-test or Wilcoxon effect size (r), with 0.10 - < 0.3 as small/negligible effect, 0.30 - < 0.5 as moderate effect, and > = 0.5 as large effect

### Postoperative anatomic outcomes

**Fig. 2:**
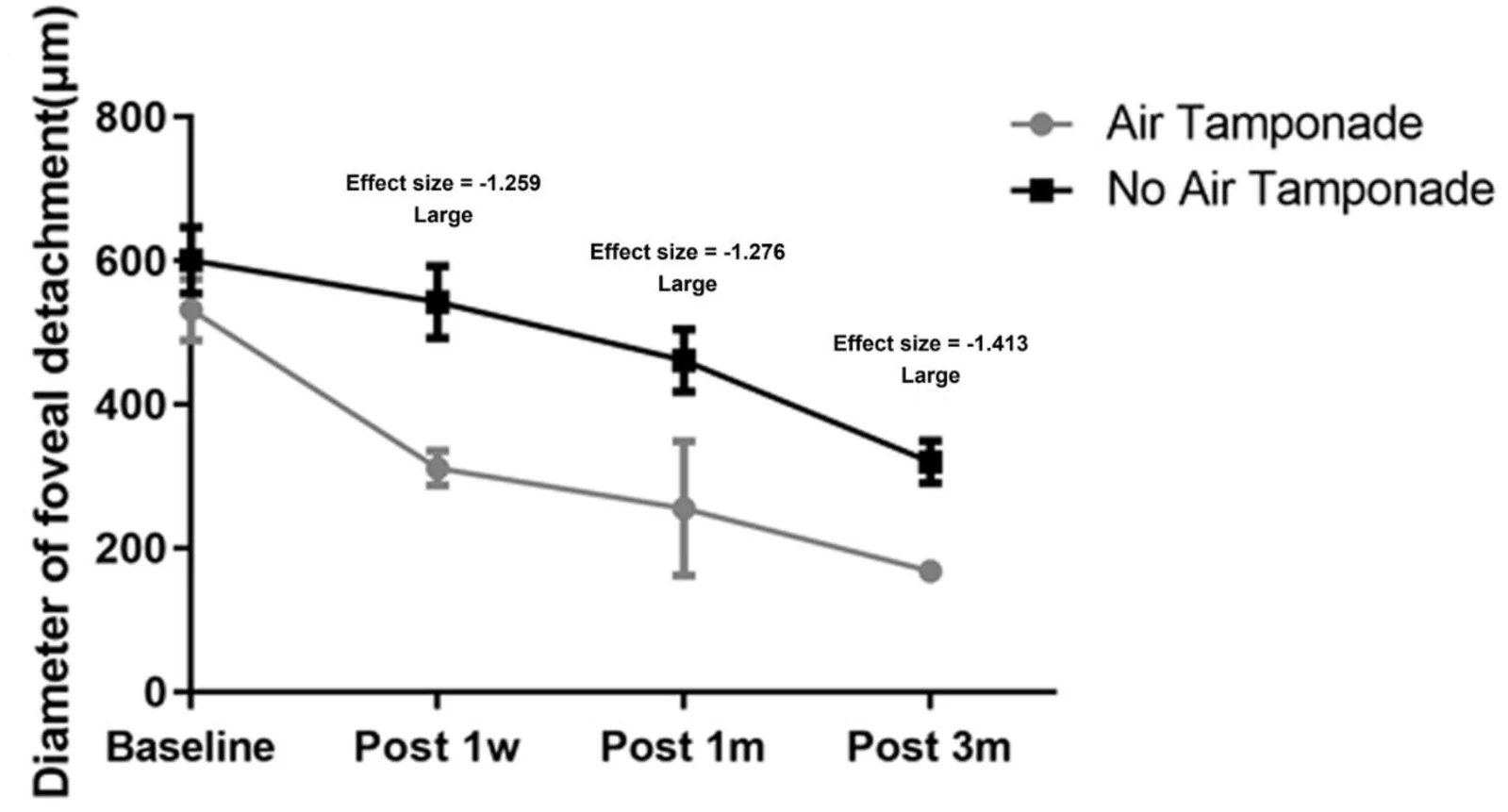
Postoperative CFT changes in both groups. Longitudinal comparison of CFT in the air-tamponade and no-air tamponade groups at preoperative baseline, 1 week, 1 month, 3 months, and the final follow-up. The air-tamponade group demonstrated significantly faster reduction in CFT at early postoperative timepoints (≤ 3 months)

**Fig. 3:**
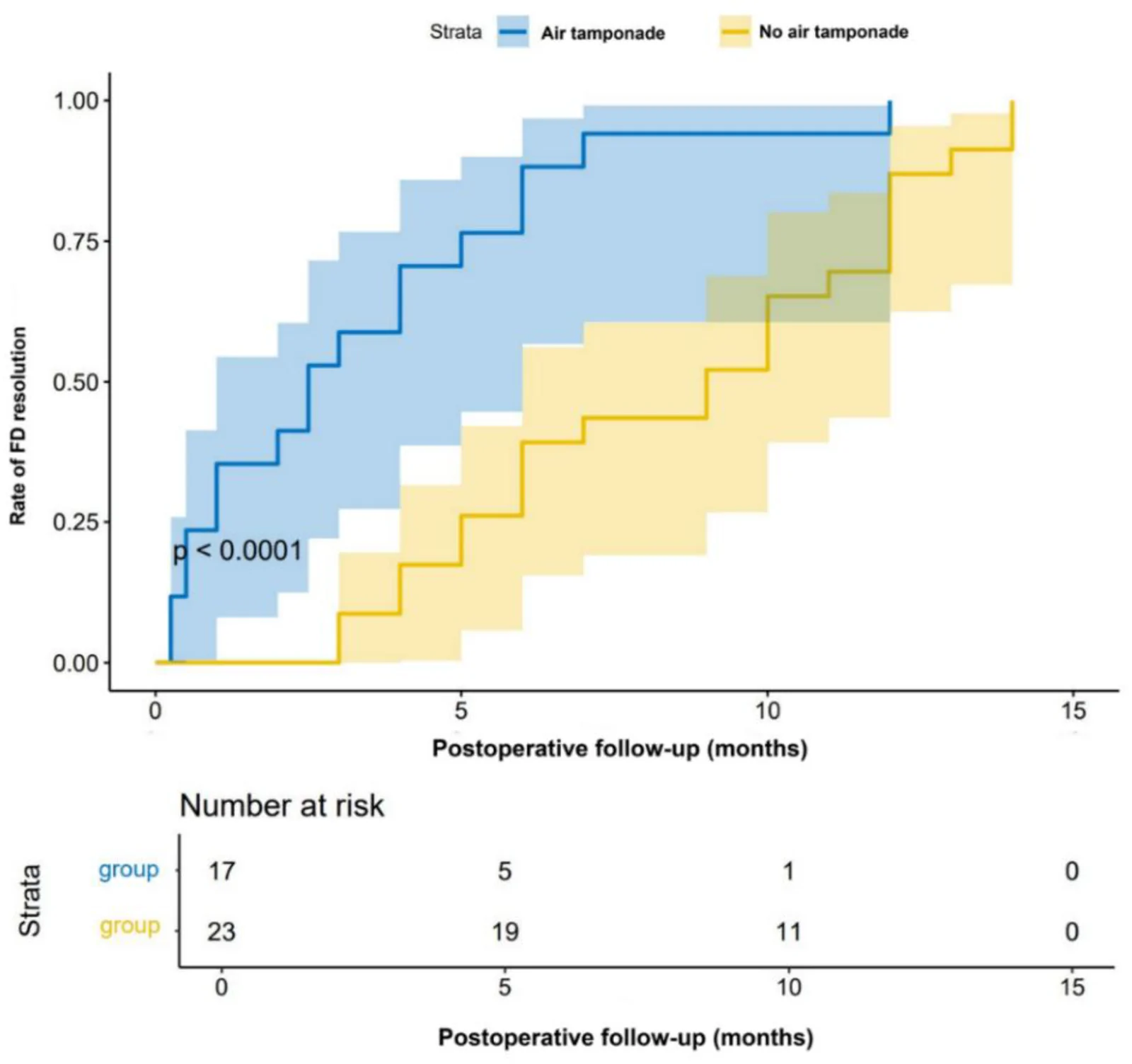
The anatomical resolution of foveal detachment (FD) in both groups. Cox proportional hazards model analysis showing time to foveal reattachment in the air-tamponade and no-air tamponade groups. The air-tamponade group exhibited a significantly faster rate of foveal detachment resolution, suggesting that intraocular air tamponade accelerates anatomical recovery. (no-air tamponade vs. air tamponade, HR: 0.2 (0.08, 0.50)

**Fig. 4:**
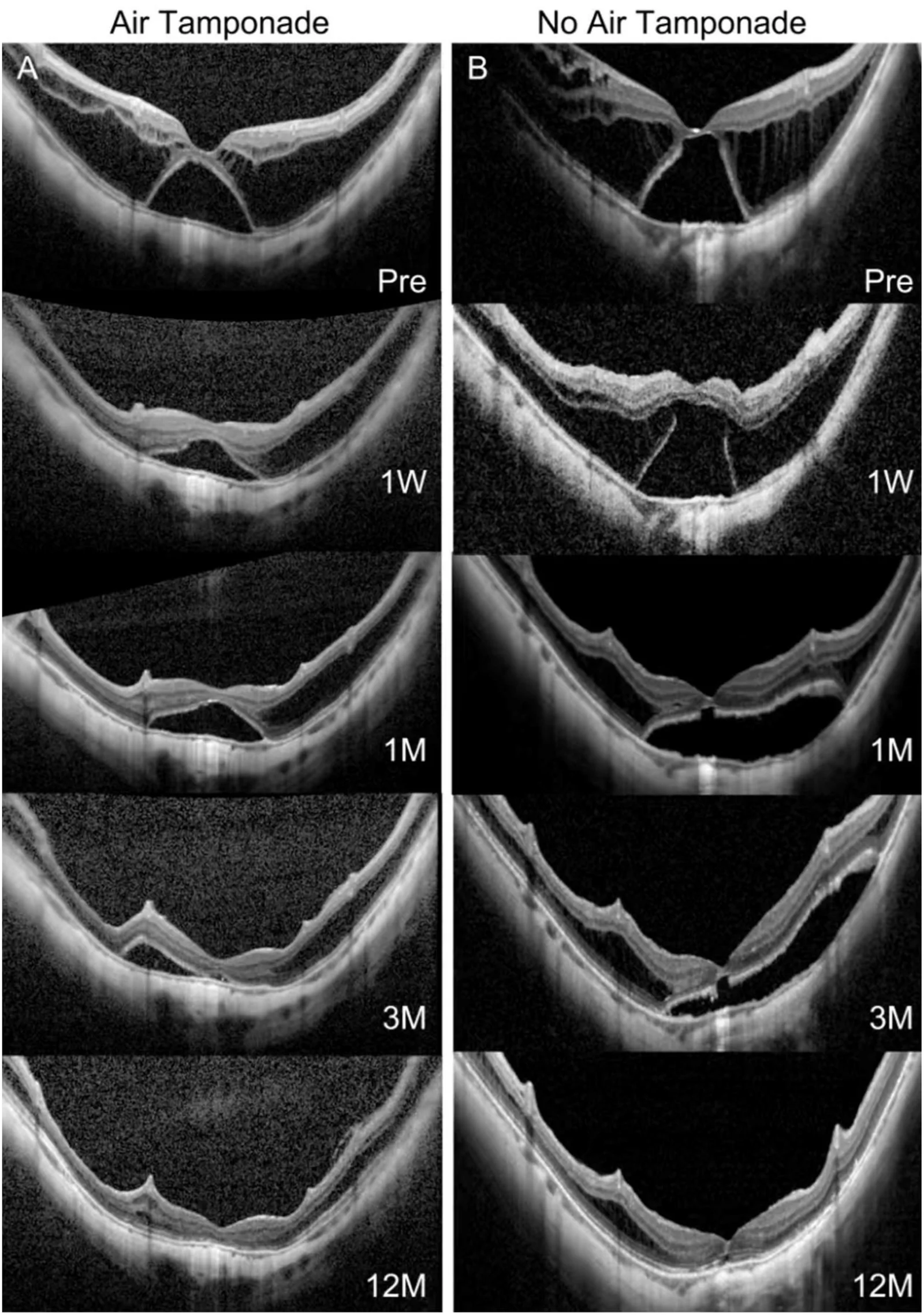
Representative OCT images of MFS eyes treated with PPV and with (**A**) or without (**B**) air tamponade. Panels show longitudinal changes at baseline (Pre), 1 week (1 W), 1 month (1 M), 3 months (3 M), and 12 months (12 M) after surgery. Compared with the no-air tamponade group, the air-tamponade group exhibited more rapid anatomical recovery of the fovea during the early postoperative period; however, final anatomical outcomes were comparable between groups

After surgery, both groups showed a reduction in the CFT over time. However, the air-tamponade group demonstrated a more rapid resolution of FD, with significantly thinner CFTs at 1 week (333.00 (282.00, 373.90) vs. 584.27 (292.00, 737.00) µm; *effect size* = -1.259; Large), 1 month (255.06 ± 93.41 vs. 460.72 ± 207.95 μm; *effect size* = -1.276; Large), and 3 months (167.34 ± 60.42 vs. 319.60 ± 139.90 μm; *effect size* = -1.413; Large) compared with the no-air tamponade group (Table 2; Fig. 2). After a 12-month follow-up, the mean CFT in the air-tamponade group (126.65 ± 40.65 μm) was lower than that in the no-air tamponade group (134.73 ± 40.70 μm), but this difference was not significant (*effect size* = -0.199; Negligible). Across most postoperative time points, the distributions of ELM and EZ integrity grades were largely comparable between the air and no-air tamponade groups, as evidenced by small effect sizes (*effect size* < 0.3), with the only exception being the ELM grade at 1 month postoperatively, which showed a moderate effect size (*effect size* = 0.477). Moreover, Fig. 3 illustrated the cumulative rate of FD resolution, demonstrating a significantly shorter time to foveal reattachment in the air-tamponade group (no-air tamponade vs. air tamponade, HR: 0.2 (0.08, 0.50); *P* < 0.001). The characteristic changes of the foveal morphology observed via OCT for the two groups are presented in Fig. 4.

**Fig. 5:**
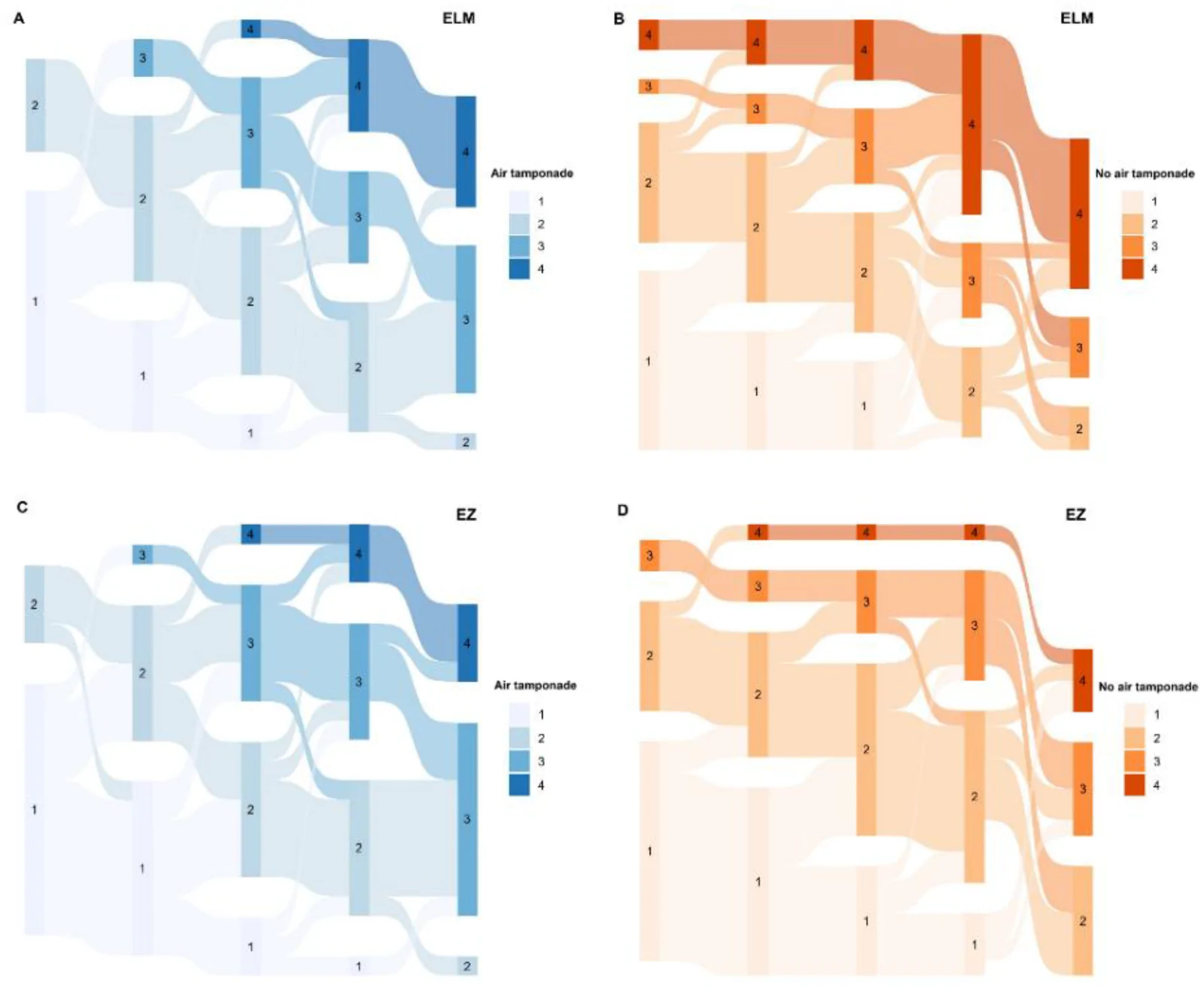
The Sankey plot of changes in outer retinal layers in both groups. The longitudinal changes in grade of ELM integrity in air tamponade group (**A**) and no-air tamponade group (**B**) after surgery; The longitudinal changes in grade of EZ integrity in air tamponade group (**C**) and no-air tamponade group (**D**) after surgery

In the longitudinal treatment (tamponade type)time interaction analysis (Table 3), for postoperative CFT value, significant larger CFT values in no-air tamponade group were detected at 1 week (estimate 162.77, 95% CI 43.11282.44, *P* = 0.008) and 1 month (estimate 137.03, 95% CI 17.36256.69, *P* = 0.025). The interaction estimates were positive at these early time points, remained non-significantly positive at 3 months (83.63, *P* = 0.17), and shifted to a non-significant negative value at 12 months (57.63, *P* = 0.373). The main effect of treatment was not significant (*P* = 0.183), whereas CFT declined from baseline at all postoperative visits (all *P* < 0.001). For ELM integrity, none of the treatmenttime interaction terms reached significance (all *P* > 0.05). For EZ integrity, significant better integrity grade in no-air tamponade group were observed at 1 month (OR 5.29, 95% CI 1.1823.65, *P* = 0.029) and 3 months (OR 5.84, 95% CI 1.1030.90, *P* = 0.038); the odds ratio at 12 months was 6.07 (*P* = 0.061) and at 1 week was 1.78 (*P* = 0.383). Figure 5 depicted the changes in ELM and EZ integrity grades after surgery, both ELM and EZ integrity grades increased gradually over time.

After adjusting for age, gender, lens status, axial length, diopter sphere, baseline BCVA, preoperative NCFT, preoperative diameter of FD, and inner retinoschisis, the multivariable mixed-effects model showed time-treatment interactions at 1 week (estimate = 192.58, *P* = 0.003) and 1 month (estimate = 166.23, *P* = 0.009) remained statistically significant (Table 4).

**Table 2:** Anatomic and visual outcomes after surgery

Characteristic	Air Tamponade	No-Air Tamponade	Effect size	
			Effect size^#^	Magnitude
**Anatomic Outcomes**				
**Postoperatively at 1 Week**	(*n* = 17)	(*n* = 23)		
CFT^2^	333.00 (282.00, 373.90)	584.27 (292.00, 737.00)	**-1.259**	**Large**
ELM Integrity (Grade 1 / 2 /3 / 4)	6 / 9 / 2 / 0	8 / 10 / 2 / 3	0.078	Small
EZ Integrity (Grade 1 / 2 /3 / 4)	9 / 7 / 1 / 0	12 / 8 / 2 / 1	0.041	Small
**Postoperatively at 1 Month**	(*n* = 17)	(*n* = 23)		
CFT^1^	255.06 ± 93.41	460.72 ± 207.95	**-1.276**	**Large**
ELM Integrity (Grade 1 / 2 /3 / 4)	2 / 8 / 6 / 1	6 / 8 / 5 / 4	0.477	Moderate
EZ (Grade 1 / 2 /3 / 4)	3 / 7 / 6 / 1	7 / 11 / 4 / 1	0.208	Small
**Postoperatively at 3 Months**	(*n* = 17)	(*n* = 23)		
CFT^1^	167.34 ± 60.42	319.60 ± 139.90	**-1.413**	**Large**
ELM Integrity (Grade 1 / 2 /3 / 4)	0 / 7 / 5 / 5	0 / 6 / 5 / 12	0.217	Small
EZ Integrity (Grade 1 / 2 /3 / 4)	1 / 7 / 6 / 3	4 / 11 / 7 / 1	0.239	Small
**Postoperatively at 12 Months**	(*n* = 15)	(*n* = 17)		
CFT^1^	126.65 ± 40.65	134.73 ± 40.70	-0.199	Small
ELM Integrity (Grade 1 / 2 /3 / 4)	0 / 1 / 8 / 6	0 / 3 / 4 / 10	0.103	Small
EZ Integrity (Grade 1 / 2 /3 / 4)	0 / 1 / 10 / 4	0 / 7 / 6 / 4	0.262	Small
**Visual Outcomes**				
Postoperative BCVA at 3 Months^2^	0.50 (0.30, 1.20) (*n* = 11)	0.75 (0.50, 1.15) (*n* = 12)	0.154	Small
Postoperative BCVA at 12 Months^2^	0.50 (0.30, 0.80) (*n* = 17)	0.40 (0.30, 1.00) (*n* = 23)	0.007	Small

Abbreviation: CFT = central fovea thickness; ELM = external limiting membrane; EZ = ellipsoid zone; BCVA = Best Corrected Visual Acuity

Statistics:

^1^Data are presented as mean ± standard deviation (SD), ^2^Data are presented as median with interquartile (IQR)

#effect size: using t-test for normally distributed continuous variables (or Wilcoxon tests for non-normally distributed continuous variables) and Wilcoxon rank-sum tests for ordinal categorical variables (ELM grades and EZ grades) for descriptive comparison, and the difference were presented with cohen’s d as the effect size for t-test or Wilcoxon effect size (r), with 0.10 - < 0.3 as small/negligible effect, 0.30 - < 0.5 as moderate effect, and > = 0.5 as large effect

**Table 3:** Longitudinal analysis of anatomic outcomes with treatment-time interaction

Predictors	CFT^1^				ELM Integrity^2^			EZ Integrity^2^		
	Estimates	CI		*P*-value^1^	Odds Ratios	CI	*P*-value^2^	Odds Ratios	CI	*P*-value^2^
**Postoperative Time Points (baseline as reference)**										
1 Week	-220.19	-310.93 -129.45		**< 0.001**	0.26	0.090.72	**0.009**	0.35	0.101.17	0.087
1 Month	-276.35	-367.08 -185.61		**< 0.001**	0.07	0.030.20	**< 0.001**	0.05	0.010.21	**< 0.001**
3 Months	-364.06	-454.80 -273.32		**< 0.001**	0.03	0.010.10	**< 0.001**	0.02	0.010.10	**< 0.001**
12 Months	-404.61	-498.79 -310.43		**< 0.001**	0.01	0.000.04	**< 0.001**	0.01	0.000.03	**< 0.001**
**Treatment (Air tamponade as reference)**										
No-Air Tamponade	68.64	-32.78170.05		0.183	0.48	0.131.75	0.266	0.48	0.121.88	0.289
**Time Points × Treatment (baseline and Air tamponade as reference)**										
1 Week: No-Air Tamponade	162.77	43.11282.44		**0.008**	1.73	0.555.43	0.346	1.78	0.496.55	0.383
1 Month: No-Air Tamponade	137.03	17.36256.69		**0.025**	2.88	0.879.53	0.083	5.29	1.1823.65	**0.029**
3 Months: No-Air Tamponade	83.63	-36.04203.29		0.17	0.88	0.164.72	0.879	5.84	1.1030.90	**0.038**
12 Months: No-Air Tamponade	-57.63	-184.9169.65		0.373	1.46	0.326.69	0.623	6.07	0.9240.05	0.061
	ICC		0.3							
	AIC		2395.719							

Abbreviation: CFT = central fovea thickness; ELM = external limiting membrane; EZ = ellipsoid zone; CI = 95% confidence interval; ICC = Interclass Correlation Coefficient; AIC = Akaike Information Criterion

Statistics:

^1^*P*-values were calculated using the mixed effect models for continuous variables; ^2^*P*-values were calculated using the Generalized Estimating Equations for categorical variables

**Table 4:** Multivariable longitudinal analysis of postoperative CFT

Predictors	Estimates	CI	*P*-value^1^
**Postoperative Time Points (baseline as reference)**			
1 Week	-250	-347.01 -152.99	**< 0.001**
1 Month	-305.55	-402.56 -208.54	**< 0.001**
3 Months	-394.1	-491.11 -297.09	**< 0.001**
12 Months	-430.49	-529.54 -331.44	**< 0.001**
**Treatment (Air tamponade as reference)**			
No-Air Tamponade	41.19	-77.28159.65	0.493
**Time Points × Treatment (baseline and Air tamponade as reference)**			
1 Week: No-Air Tamponade	192.58	67.89317.28	**0.003**
1 Month: No-Air Tamponade	166.23	41.54290.93	**0.009**
3 Months: No-Air Tamponade	113.66	-11.03238.36	0.074
12 Months: No-Air Tamponade	-29.24	-160.58102.10	0.661
Age(y)	-1.89	-6.152.38	0.384
Gender	3.04	-93.0099.07	0.95
Lens status	-12.61	-188.55163.33	0.888
Axial Length (mm)	6.97	-36.2450.18	0.75
Diopter Sphere (D)	0.39	-16.5917.37	0.964
Baseline BCVA (LogMAR)	-8.14	-73.6557.36	0.806
Preoperative NCFT (µm)	-0.07	-0.460.32	0.72
Preoperative diameter of foveal detachment (µm)	0.05	-0.030.13	0.233
Preoperative inner retinoschisis	-19.86	-139.6099.87	0.744
ICC	0.3		
AIC	2395.719		

Abbreviation: BCVA = Best Corrected Visual Acuity; NCFT = neurosensory central foveal thickness; CFT = central fovea thickness; ELM = external limiting membrane; EZ = ellipsoid zone; CI = 95% confidence interval; ICC = Interclass Correlation Coefficient; AIC = Akaike Information Criterion

Statistics:

Longitudinal analyses were calculated using the mixed effect models

^1^*P*-values for multivariable analysis, variables including age, gender, lens status, axial length, diopter sphere, preoperative BCVA, preoperative diameter of foveal detachment (µm), preoperative inner retinoschisis and preoperative NCFT were also entered the mixed effect model to adjust the effects of tamponade type on BCVA at 12 months at different time points

### Postoperative visual outcomes

At 3 months and 12 months, no significant differences in the mean postoperative BCVA (LogMAR) were detected between the air-tamponade group and the no-air tamponade group (both *effect size* < 0.3; Small) (Table 2). In the longitudinal mixed-model analysis (Table 5), postoperative BCVA (LogMAR) improved significantly from baseline at both 3 and 12 months (all *P* < 0.01). The main effect of tamponade type (no-air tamponade vs. air tamponade) was not statistically significant in either univariable or multivariable models. In the univariable analysis, a significant time-by-treatment interaction emerged at 12 months (estimate: −0.32, 95% CI: −0.61 to − 0.03, *P* = 0.03); eyes without air tamponade had an additional reduction of 0.32 LogMAR BCVA relative to the air-tamponade group. This interaction was not observed at 3 months (*P* = 0.301). After adjusting for age, gender, lens status, axial length, diopter sphere, preoperative CFT, preoperative NCFT, preoperative diameter of FD, and inner retinoschisis, preoperative ELM / EZ integrity, and preoperative diameter of FD, the time-by-treatment interaction at 12 months was attenuated and no longer statistically significant (multivariable estimate: −0.27, 95% CI: −0.57 to 0.03, *P* = 0.078). Among the covariates, only a larger preoperative diameter of FD was significantly associated with worse BCVA at 12 months (per µm: estimate: 0.00, 95% CI: 0.00 to 0.00, *P* = 0.004; per 500-µm: estimate: 0.23, 95% CI: 0.09 to 0.37, *P* = 0.004).

**Table 5:** Longitudinal analysis of BCVA with treatment-time interaction

Predictors	Univariable Longitudinal Analysis			Multivariable Longitudinal Analysis for BCVA at 12 months		
	Estimates	CI	*P*-value^1^	Estimates	CI	*P*-value^2^
**Postoperative Time Points (baseline as reference)**						
3 Months	-0.42	-0.67 -0.16	**0.002**	-0.48	-0.75 -0.21	**0.001**
12 Months	-0.42	-0.64 -0.20	**< 0.001**	-0.47	-0.70 -0.23	**< 0.001**
**Treatment (Air tamponade as reference)**						
No-Air Tamponade	0.25	-0.150.66	0.213	0.33	-0.080.74	0.111
**Time Points × Treatment (baseline and Air tamponade as reference)**						
3 Months: No-Air Tamponade	-0.18	-0.540.17	0.301	-0.13	-0.490.24	0.497
12 Months: No-Air Tamponade	-0.32	-0.61 -0.03	**0.03**	-0.27	-0.570.03	0.078
Age(y)				0.02	-0.000.03	0.087
Gender				-0.23	-0.660.19	0.28
Lens status				-0.33	-1.070.42	0.385
Axial Length (mm)				0.06	-0.130.24	0.545
Diopter Sphere (D)				0.04	-0.030.10	0.303
Preoperative inner retinoschisis				-0.02	-0.550.51	0.94
Preoperative NCFT (µm)				0	-0.000.00	0.412
Preoperative diameter of foveal detachment (µm)				0	0.000.00	0.004
Preoperative diameter of foveal detachment (per 500-µm)				0.23	0.090.37	**0.004**
Preoperative CFT (µm)				0	-0.000.00	0.892
Preoperative ELM integrity				-0.33	-2.251.58	0.729
Preoperative EZ integrity				-0.36	-2.071.34	0.675
ICC	0.75			0.59		
AIC	164.94			181.24		

Abbreviation: BCVA = Best Corrected Visual Acuity; NCFT = neurosensory central foveal thickness; CFT = central fovea thickness; ELM = external limiting membrane; EZ = ellipsoid zone; CI = 95% confidence interval; ICC = Interclass Correlation Coefficient; AIC = Akaike Information Criterion

Statistics:

Longitudinal analyses were calculated using the mixed effect models

^1^*P*-values for univariable analysis of the effects of tamponade type on postoperative BCVA at different time points

^2^*P*-values for multivariable analysis, variables including age, gender, lens status, axial length, diopter sphere, preoperative diameter of foveal retinal detachment (µm), preoperative inner retinoschisis, preoperative CFT, preoperative NCFT and preoperative ELM/EZ integrity were also entered the mixed effect model to adjust the effects of tamponade type on BCVA at 12 months at different time points

### Postoperative adverse events

Postoperative IOP values at both 1 day and 1 month did not significantly differ between the two groups. In terms of postoperative complications, transient aggravation, defined as temporary worsening of macular morphology (Supplemental Fig. 2), was more frequent in the no-air tamponade group (30.8%) than in the air tamponade group (9.5%), and temporary choroidal leakage was observed in the no-air tamponade group (2 of the 26 eyes, 7.7%). However, no statistically reliable differences between the two groups for both events were detected, which the odds ratios had very wide 95% confidence intervals that included 1. The two eyes with temporary choroidal leakage finally achieved anatomical recovery and visual improvement. Eyes that received air tamponade developed postoperative MH in 4 cases (19.0%), compared with 3 cases (11.5%) in those without tamponade, among these, MH-associated RD was observed in 2 eyes (9.5%) and 1 eye (3.8%), respectively (Table 6). However, the 95% confidence intervals for both estimates were extremely wide and included 1, presenting no statistically reliable differences between the two groups. The preoperative characteristics, therapy and visual outcomes were summarized in Table 7, two patients were followed-up with stable anatomical structure and therefore they refused reoperation, other patients underwent reoperation and all achieved anatomical recovery.

**Table 6:** The surgical adverse events with or without air tamponade

Characteristic^1^	Air Filling Status					
	Air Tamponade *N* = 21	No-Air Tamponade *N* = 26	Overall *N* = 47^1^	Effect size		
				*Effect size* ^#^	Magnitude	
Postoperative IOP at 1 day (mmHg)	12.00 (7.70, 14.00)	8.00 (7.00, 10.00)	9.00 (7.00, 13.00)	0.282	Small	
Postoperative IOP at 1 month (mmHg)	15.38 ± 3.03	15.57 ± 3.16	15.30 ± 3.03	0.086	Small	
Postoperative MH	4 (19.0%)	3 (11.5%)	7 (15%)	1.97 (0.20, 25.92)		
Postoperative MH-retinal detachment	2 (9.5%)	1 (3.8%)	3 (6.4%)	1.24 (0.02, 101.91)		
Transient aggravation	2 (9.5%)	8 (30.8%)	10 (21%)	0.24 (0.02, 1.45)		
Postoperative choroidal leakage	0 (0.0%)	2 (7.7%)	2 (4.3%)	0.00 (0.00, 6.58)		

Abbreviation: MH = Macular Hole

^1^Data are presented as mean ± standard deviation (SD), ^2^Data are presented as median with interquartile (IQR)

#effect size: using t-test for normally distributed continuous variables (or Wilcoxon tests for non-normally distributed continuous variables) and Chi-squared test (or Fisher exact test) for descriptive comparison, and the difference were presented with Cohen’s d as the effect size for t-test or Wilcoxon effect size (r), with 0.10 - < 0.3 as small/negligible effect, 0.30 - < 0.5 as moderate effect, and > = 0.5 as large effect. Odds ratios (ORs) with 95%CI were applied for the evaluation of the effect size of categorical comparison, and OR with a 95%CI not overlap with 1 were considered significant magnitude

**Table 7:** Clinical characteristics and treatment outcomes of patients with post-operative MH or MH-RD

No.	Tamponade	Sex	Age (y)	Baseline BCVA (LogMAR)	Axial length (mm)	Diopter Sphere	Lens status	Preoperative Diameter of FD (µm)	Preoperative CFT (µm)	ELM / EZ status	Interval to MH or MH-RD diagnosis	Further therapy	BCVA at 12 months (LogMAR)	Anatomic outcome
1	Air tamponade	M	52	1.3	29.88	-12.0	Phakic	919.19	438.93	1/1	1week, MH-RD	PPV + SO tamponade*1	1	RD recovery MH closure
2	Air tamponade	F	62	0.9	30.05	-14	Pseudophakic	729.22	557.13	1/1	1week, MH-RD	PPV + SO tamponade*1	1	RD recovery MH closure
3	Air tamponade	M	52	2.2	31.75	-18	Phakic	1900.38	975.00	1/1	1week, MH	PPV + SO tamponade*1	1.7	MH closure
4	Air tamponade	F	51	0.7	29.75	-16	Phakic	1604.15	331.73	1/1	6 months, MH	Follow-up	0.5	Stable
5	No-Air tamponade	M	48	1.3	26.67	-8.5	Phakic	580.42	727.842	1/1	1 month, MH-RD	Follow-up	1.3	Stable
6	No-Air tamponade	F	74	0.9	31.15	-6.0	Pseudophakic	1180.87	924.06	1/1	1 week, MH	PPV+C3F8 tamponade*1	0.7	MH closure
7	No-Air tamponade	F	58	1.7	30.06	-17.0	Phakic	957.38	614.89	2/2	1 month, MH	PPV + SO tamponade*1	1.3	MH closure

MH = macular hole; RD = retinal detachment; BCVA = best-corrected visual acuity; M = male; F = female; ELM = external limiting membrane; EZ = ellipsoid zone; PPV = pars plana vitrectomy; SO = silicone oil

## Discussion

PPV combined with fovea-sparing ILM peeling and long-acting gas tamponade is considered the standard surgical approach for MFS. However, the benefits of tamponade usage are debated. Some studies have shown that long-acting gas tamponade is a sufficient treatment for MTM [25, 26], whereas others have reported successful treatment of MFS without tamponade [27, 28]. A recent study found that eyes receiving long-acting gas tamponade (C_3_F_8_, SF_6_) achieved significantly faster resolution of MFS, but led to poorer visual outcomes compared to no tamponade [23]. It was assumed that long-acting gas tamponade could exert mechanical burden on the fragile fovea with MFS, leading to structure thinning and a higher risk of vision-threatening complications like postoperative MH. These concerns raised the question of whether air tamponade could promote anatomical recovery without inducing the risks linked to long-acting gas tamponade. Accordingly, the present study was designed to investigate the role of short-acting air tamponade in managing FD, with the aim of facilitating its resolution while minimizing potential gas-related damage. Herein, we compared the anatomical and functional outcomes, as well as the postoperative complications of undergoing PPV with and without air tamponade for MFS and FD eyes, and the preoperative risk factors of visual outcome were also analyzed.

Anatomic recovery was ultimately achieved in both the air-tamponade and no-air tamponade groups. In the early postoperative phase, the use of air tamponade was linked to a more rapid re-establishment of the central fovea, an observation that aligned with prior study using long-acting gases [23]. This difference in the speed of anatomic recovery diminished over time, with both groups exhibiting comparable anatomical outcomes by the 12-month follow-up. The acceleration of retinal reattachment in the air-tamponade group supports the rationale for using air tamponade in cases where rapid restoration of foveal morphology is clinically desirable. Moreover, the ELM integrity demonstrated improvement without treatment interaction effects. The no-air tamponade group showed significantly higher odds of intact EZ at postoperative months 1 and 3. However, no significant difference was observed at month 12. These findings suggested that omitting air tamponade may better preserve outer retinal integrity only at early postoperative follow-up.

However, these early anatomical benefits in no-air tamponade did not translate into a robust functional gain after comprehensive adjustment; the univariable visual acuity advantage at 12 months was attenuated and no longer significant in the multivariable model. Previous studies found that final postoperative visual acuity has been positively associated with preserved retinal thickness [29, 30]. And the use of tamponade may be associated with macular atrophy. [31]. Given that the retinas in high myopic eyes are characteristically thin [32], they may exhibit reduced tolerance to mechanical pressure of gas, potentially predisposing them to pressure-induced retinal damage. Moreover, a structural disorganization within the retina, previously described as “schisis bending”, has been observed in the eyes receiving long-acting gases tamponade [23], which might contribute to impaired visual acuity outcome. In the present study, the CFT value, ELM and EZ integrity at 12 months in eyes with air tamponade was not significantly different with eyes without air tamponade, which was corresponding to the comparable BCVA at 12 months. These findings could be explained by the milder and shorter-lived mechanical pressure of air tamponade on the fovea compared to that of long-acting gases, minimizing the mechanical damage to the fovea. To validate our results, future randomized prospective studies with larger samples are required.

In the multivariable mixed-effects model, the only influential risk factor of worse 12-month BCVA was a larger preoperative FD diameter (*P* = 0.004). When measured per-micrometer, the estimates and 95% CI both round to 0.00, this may reflect the tiny per-micrometer effect of FD. However, scaled to a clinically meaningful unit, each 500‑µm increase in preoperative FD diameter was independently associated with a 0.23‑logMAR worsening in 12‑month BCVA (95% CI: 0.09 to 0.37, *P* = 0.004). Given the wide clinical range of FD, this effect size translates into a substantial cumulative impact on final visual acuity. The attenuation of the univariable treatmenttime interaction on BCVA after adjustment further indicated that baseline anatomical severity, not tamponade strategy, may primarily drive the long-term visual function. Further prospective studies with extended follow-up and larger sample are warranted to clarify whether omitting air tamponade could ultimately provide functional benefits.

The development of postoperative full-thickness MH or MH-related RD is a serious vision-threatening complication and is difficult to treat, with reported incidences ranging from 5% to 27.3% [14–17]. MFS complicated by FD with inner retina thinning has been indicated as a strong risk factor for postoperative MH formation. The role of gas tamponade in formation of postoperative MH remains controversial. It was concerned that its mechanical pressure may rupture the vulnerable fovea, inducing surgery-related MH [22]. Conversely, it was hypothesized that facilitating faster retinal reattachment via gas tamponade could improve choroidal oxygen diffusion to the retina and thereby reduce MH risk—a mechanism aligned with its proven efficacy in MH repair [33].

Therefore, we attempted to refine the surgical protocol for eyes exhibiting MFS with FD to reduce the risk of postoperative MH formation. The technique of FSIP, previously demonstrated to be protective for postoperative MH [12, 34], was conducted in all eyes in the present study. Nevertheless, postoperative MH or MH-RD still occurred in both the air-tamponade and no-air tamponade groups. In our study, the observed odds ratios for postoperative MH and MH-RD were higher in the air tamponade group than in the no-air tamponade group, but the extremely wide 95% confidence intervals that included 1 precluded any statistically reliable conclusion. Thus, no statistically reliable difference was detected between the two groups regarding postoperative MH or MH‑RD formation, consistent with previous reports using long-acting gases [23]. However, owing to the limited sample size, this study was underpowered to detect differences in postoperative MH occurrence, raising the possibility of a Type II error. The numerically higher absolute rates (MH: 19.0% vs. 11.5%; MH-RD: 9.5% vs. 3.8%) warranted concerns and highlighted the need for larger, adequately powered prospective studies to better characterize the safety profile of air tamponade.

Although the differences did not reach statistical significance, transient aggravation was observed more frequently in the no-air tamponade group, and choroidal leakage occurred only in the no-air tamponade group. These findings suggest a protective effect of air tamponade, possibly providing immediate mechanical support to the fovea and stabilizing retinal architecture during the critical early postoperative period. Nevertheless, all cases of transient aggravation and choroid leakage resolved spontaneously without additional intervention, and our visual outcome data indicated that neither complication had a detrimental impact on the long-term visual prognosis.

This study has several limitations. First, owing to the challenges of conducting randomized prospective studies in patients with MFS combined with FD in real-world settings, the investigation was designed as a retrospective analysis with strict inclusion criteria to ensure baseline comparability and minimize selection bias, finally, 47 eyes were enrolled for analysis and this relatively small sample size of this study may increase the risk of Type II errors, especially for complications such as postoperative MH, as the statistical power may be insufficient to detect between‑group differences. In this retrospective study, the sequential, calendar‑period allocation of treatment (air tamponade from 2017 to 2019, no‑air tamponade thereafter) could introduce potential confounding by temporal trends, including changes in surgical experience, instrumentation, postoperative follow-up, or clinical decision-making. The observed outcomes may be influenced by these time‑varying factors rather than the tamponade agent itself. Future large-scale, perspective, randomized, multicenter studies are therefore warranted. Additionally, incomplete postoperative follow-ups for some patients led to missing data at certain timepoints. Although the missing data were consistent with missing at random and a mixed‑effects model was used to handle missing data, the impact of loss‑to‑follow‑up bias on the interpretation of effect estimates and long‑term outcomes cannot be completely excluded. To address this, multivariate mixed-effects models was employed to eliminate these potential biases. Another limitation was that our longitudinal analyses were performed on the PP set, excluding eyes that developed postoperative MH or MH‑RD—a subset with the poorest anatomical and functional prognoses. This exclusion may introduce bias, potentially leading to overestimation of CFT and BCVA improvements in the remaining cohort. An intention‑to‑treat analysis would be preferable but was not possible due to the retrospective design and because these events could not be included in the longitudinal analysis.

## Conclusion

In summary, air tamponade accelerated early anatomical recovery in highly myopic eyes with MFS and FD. However, the long‑term anatomical outcomes, as well as both visual outcomes, were comparable between the air tamponade and non‑air tamponade groups. Larger preoperative diameter of FD was identified as the independent risk factor of worse visual outcomes at 12 months. No statistically reliable difference was detected between the two groups regarding postoperative MH or MH‑RD formation.

## Supplementary Information

Below is the link to the electronic supplementary material.

Supplementary Material 1: Supplemental Fig. 1. Representative OCT images illustrating the grading of ELM and EZ integrity after surgery. A) Case (1) One week postoperatively, the ELM and EZ remain absent at the central fovea (ELM grade 1; EZ grade 1). B) Case (2) One week postoperatively, both the ELM and EZ bands are present but still show broad disruption > 200 μm (ELM grade 2; EZ grade 2). C) Case (3) The ELM and EZ are partially restored, with focal interruption < 200 μm (ELM grade 3; EZ grade 3). D) Case (4) Both the ELM and EZ appear continuous at the central fovea (ELM grade 4; EZ grade 4)

Supplementary Material 2: Supplemental Fig. 2. The classical features of postoperative temporary choroidal leakage. One week after PPV with no-air tamponade, wavy changes of the choroid and retina on postoperative OCT images were observed, accompanied by unclear refractive media due to the leakage. The wavy changes of neuroretina and unclear refractive media alleviated along with time, with FD recovery. At 12 months, part of protruding changes of RPE persisted

## Data Availability

All data analyzed or generated in this study are included in the article. Further reasonable requests for these data are available from the corresponding authors.

## Abbreviations

MTM: Myopic traction maculopathy
MFS: Myopic foveoschisis
FD: Foveal detachment
MH: Macular hole
PPV: Pars plana vitrectomy
ILM: Internal limiting membrane
FSIP: Fovea-sparing internal limiting membrane peeling
SF6: Sulfur hexafluoride
C_3_F_8_: Perfluoropropane
RD: Retinal detachment
BCVA: Best-corrected visual acuity
logMAR: Logarithms of the minimum angle of resolution
IOP: Intraocular pressure
OCT: Optical coherence tomography
CFT: Central foveal thickness
NCFT: Neurosensory central foveal thickness
ELM: External limiting membrane
EZ: Ellipsoid zone
